# Peripartal changes of metabolic and hormonal parameters in Romanian spotted cows and their relation with retained fetal membranes

**DOI:** 10.3389/fvets.2024.1409666

**Published:** 2024-05-23

**Authors:** Horatiu Rafa, Ioan Oroian, Oana Maria Cozma, Andreea Georgiana Morohoschi, Daria Antonia Dumitraș, Cristina Laura Ștefănuț, Daniela Neagu, Alex Borzan, Sanda Andrei

**Affiliations:** ^1^Department of Preclinical Sciences, Faculty of Veterinary Medicine, University of Agricultural Sciences and Veterinary Medicine Cluj-Napoca, Cluj-Napoca, Romania; ^2^Cattle Breeding Research and Development Station, Sângeorgiu de Mureş, Romania; ^3^Department of Clinical Sciences, Faculty of Veterinary Medicine, University of Agricultural Sciences and Veterinary Medicine Cluj-Napoca, Cluj-Napoca, Romania

**Keywords:** cows, parturition, placenta, metabolic profile, hormones

## Abstract

This clinical study investigates various metabolic and physiological parameters in dairy cows during puerperium. Retained fetal membranes (RFM) is a significant postpartum complication that can affect the overall health, fertility and productivity of dairy cattle. The research focuses on changes in total proteins, albumin, glucose, triglycerides, total cholesterol, aspartate aminotransferase (AST) and alanine aminotransferase (ALT), cortisol, insulin, and insulin-like growth factor 1 (IGF-1) levels among cows experiencing normal post-partum period (NP) and those with RFM. A significant increase in protein levels was noted during the post-partum period in the RFM group, indicating physiological impacts of RFM at this stage. Albumin levels showed significant differences, highlighting a significant biological effect of RFM in the post-partum period. Glucose levels varied significantly in the weeks leading to parturition, suggesting altered metabolic states in cows that suffered RFM. Triglyceride and cholesterol levels were significantly higher during the antepartum period in the group that experienced reproductive failure, indicating substantial alterations in lipid metabolism which could herald the apparition of RFM. AST and ALT levels provided insights into cellular stress and liver function, with significant increases noted around parturition which could be attributed to the substantial physiological strain of parturition itself. Cortisol levels were higher in RFM cows 2 weeks before parturition, which could indicate an increasing stress response or a physiological preparation for the upcoming labor, and may be more pronounced in cows predisposed to RFM. Insulin levels decreased significantly before and at parturition in RFM cows, indicating a strong energy deficit. IGF-1 levels decreased significantly in RFM cows after parturition. Significant changes in metabolic parameters, such as glucose, triglycerides, and cholesterol levels, delineate the pronounced metabolic challenges faced by cows with RFM. The study elucidates that while some variations are noted as parturition approaches, the most substantial impacts attributable to RFM on metabolic and physiological parameters occur after parturition. These changes may have implications for the health, recovery, and productivity of cows postpartum, suggesting the need for targeted management strategies to mitigate the effects of RFM.

## 1 Introduction

The study of postpartum uterine diseases occupies a crucial position in veterinary medical research, impacting not only the welfare of animals but also their reproductive efficiency. These conditions lead to a decline in reproductive function, which is associated with increased financial burdens, manifested through decreased milk production and higher costs of medical treatment (1). Among such conditions, retained fetal membranes (RFM) is particularly significant, as it prevents the normal process of uterine involution and contributes to the development of chronic endometritis, both during and after the puerperal period, culminating in diminished fertility (2, 3).

Endocrinologically, the period of parturition is characterized by the intricate interplay of a variety of biologically active substances, including steroidal and non-steroidal hormones, along with prostaglandins (4). The transitional phase, which spans from the end of gestation to the commencement of lactation, represents the most metabolically challenging period for dairy cows, necessitating a complex metabolic adaptation that involves the activation and coordination of metabolic pathways. This adaptation is influenced by endocrine and neuroendocrine systems in response to environmental and physiological changes, thereby affecting the metabolism of proteins, lipids, and carbohydrates across various tissues (5).

To satisfy the increased endogenous demands for energy and nutrients, there is an enhancement in hepatic gluconeogenesis and the mobilization of proteins and lipids within the body (5). A deficiency in prepartum diet can lead to reduced serum glucose and insulin levels, instigating adipose tissue lipolysis, an increase in serum unsaturated free fatty acids, accumulation of hepatic triglycerides, and consequently, the onset of steatosis and ketosis. Addressing the negative energy balance is deemed crucial for the prevention of fatty liver conditions (6, 7). Consequently, there is a significant correlation between fatty liver syndrome and ketosis. Typically, fatty liver syndrome is concomitantly linked with liver function irregularities. These irregularities manifest as a diminished oxidation of non-esterified fatty acids within the tricarboxylic acids cycle and an impaired synthesis of lipoproteins. As a result, there is a rapid and sustained elevation in the plasma concentrations of ketone bodies (8).

Biochemical markers such as total protein (TP) and albumin (ALB) are indicators of liver synthetic function, as they are produced solely by hepatocytes. Decreases in these markers suggest reduced hepatic synthesis. Additionally, enzymes like aspartate aminotransferase (AST) and alanine aminotransferase (ALT), serve as biomarkers for hepatocyte damage; elevated levels in the blood indicate hepatocellular injury or necrosis. Thus, analyzing these enzyme activities provides insight into liver health (8).

The maternal immune system’s recognition of class I proteins of the major histocompatibility complex within a mature placenta initiates immune and inflammatory responses that are crucial for the expulsion of the allantochorion at parturition. This underscores the immune system’s broader role beyond mere pathogen recognition. The ensuing maternal immune response, which is characterized by the production of leukocyte activation factors, plays a key role in the immunological detachment of the allantochorion. A decrease in the activity of peripheral leukocytes is linked to an increased incidence of placental retention and related complications (9).

Moreover, Insulin-like growth factor 1 (IGF-I) significantly influences the immune response across different animal species, including cattle (10), by affecting the population of T helper 1 lymphocytes (11). These lymphocytes are essential to the immune mechanism responsible for the elimination of the placenta in the postpartum period (12). Cortisol, an immunosuppressive hormone, inhibits the proliferation and vital functions of leukocytes, thereby disrupting the normal immunological processes for identifying and eliminating fetal tissues (7, 13). Insulin contributes to the regulation of glucose supply to various tissues, including the uterine smooth muscles, thus playing a crucial role in regulating uterine motility and the strength of contraction (7, 14).

The incidence of RFM can be influenced by various factors, including metabolic and hormonal changes around parturition. Understanding these changes in Romanian Spotted cows, a breed significant to the Romanian dairy industry, is crucial for developing better management and intervention strategies to minimize the incidence of RFM and improve animal health and productivity.

The goals of this study are to characterize the metabolic (cholesterol, triglycerides, glucose, protein, albumin) enzymatic (ASAT, ALAT) and hormonal (insulin, cortisol, IGF-1) profiles of Romanian Spotted cows during the peripartum period, and to explore the dynamics of these metabolic and hormonal changes before, during, and after parturition and their possible correlations with RFM.

This study is among the first to focus on the Romanian Spotted breed, offering valuable breed-specific data that can inform targeted interventions and management practices.

By examining a wide range of metabolic and hormonal parameters over a nine-week period encompassing pre-parturition, parturition, and post-parturition, this study provides a detailed temporal understanding of the physiological changes occurring around calving.

The comparison of metabolic and hormonal profiles between cows with and without RFM may identify novel predictors or biomarkers for RFM, contributing to early detection and prevention strategies. Insights from this study could lead to the development of nutritional or management interventions tailored to the specific needs of Romanian Spotted cows during the peripartum period, ultimately enhancing animal welfare and productivity.

## 2 Materials and methods

### 2.1 Chemicals and reagents

The kits utilized in the current study were procured from Elabscience Biotechnology Inc., located in Texas, HT, USA. The additional chemicals were acquired from Sigma Aldrich and Merck, both located in Darmstadt, Germany.

### 2.2 Experimental animals

The clinical study involved a collaboration with a Romanian Spotted cattle farm located in Mureș County, Romania. At that time, the farm housed a total of 240 cattle, including both adults and young stock. The study was conducted between February 2021 and October 2021, during which a group of 50 cows was identified for which the breeding date and approximate calving date were verified.

The Bălțată Românească (Romanian Spotted, RS) breed, classified under the Simmental group, serves a bi-functional purpose and is currently documented with a population of 376,000 cows, comprising 36% of Romania’s cattle breed demographic (15, 16). Originating in the 18th century, the RS breed was developed through the non-methodical interbreeding of Simmental bulls, imported from Austria and Switzerland, with domestically undeveloped Podolic Grey cattle (16, 17). The lactational milk yield of the RS breed is quantified between 5,000 and 5,700 kg, with the adult female’s body mass ranging from 600 to 620 kg and the fattening young bulls experiencing an average daily weight gain of 1,000 to 1,200 g (16, 18). The breeding selection index for the RS is strategically concentrated on enhancing milk production (50%), with additional emphasis on the improvement of growth rates and carcass quality (20%), as well as the augmentation of fitness-associated characteristics (30%) (16, 19).

With regards to the shelter infrastructure, it features walls enclosing three sides, leaving one elongated side completely open. The construction material for these walls consists of reinforced concrete elements. The roofing design incorporates a single-sloped configuration, with the lowest point situated at 1.6 meters in the rear section of the structure, while the highest point reaches 3.2 meters toward the front. The flooring substrate is composed of concrete, providing an optimal foundation for the application of a continuous bedding system. This bedding is replenished with straw biweekly and replaced every 6–7 weeks or as dictated by specific circumstances. The shelter facility is equipped with two calving enclosures, each boasting a 20-square meter area, facilitating the segregation of cattle approaching the calving phase. Post-calving, cows are housed within these enclosures alongside their calves, effectively mitigating potential conflicts and injuries.

### 2.3 Experimental model

The study conducted on a total of 50 cows involved multiple samplings, both during the ante and post-partum periods, following the protocol below:

- Antepartum period: at 4, 3, 2, 1 week(s) before parturition (AP-W4; AP-W3; AP-W2; AP-W1)
- At the moment of parturition ±12 h after parturition (P)
- Postpartum period: 1, 2, 3, 4 weeks after parturition (PP-W1; PP-W2; PP-W3; PP-W4)

Blood samples were collected at the same period of time, between 6 and 8 am, all through the course of the experiment, from the coccygeal vein using vacutainers with a coagulant. After collection, they were left at room temperature to allow serum expression. The respective serum was separated and placed in Eppendorf tubes, each tube containing 0.5 mL of serum. The samples were stored at −80°C.

From the initial group of 50 cows, only 22 met the criteria for inclusion in the study by providing the necessary nine samples for evaluation. Throughout the experiment, situations of abortion, misdiagnosed pregnancies, or premature births before the calculated term were encountered. At the end of the experiment, it was observed that out of the 22 cows, only 7 presented placental retention, while the remaining cattle had no postpartum complications.

All applicable international, national and institutional guidelines for the care and use of animals were followed. The animal study protocol was ethically approved by the university local Bioethics Committee (no.417/13.12.2023) and followed the guidelines of the European Law Directive 63/2010, materialized by Romanian National Law no. 43/2014.

### 2.4 Biochemical analysis

Various parameters including albumin (ALB), glucose (GLU), triglycerides (TRG), cholesterol (COL), aspartate aminotransferase (AST)/glutamic oxaloacetic transaminase (GOT) and alanine aminotransferase (ALT)/glutamic-pyruvic transaminase (GPT) and total protein were quantified in the serum samples employing specific assay kits (Elabscience Biotechnology Inc., Texas, HT, USA). All biochemical analyses underwent rigorous validation and were performed through spectrophotometry using the SPECTROstar® Nano microplate spectrophotometer, BMG Labtech in Germany.

### 2.5 Determination of hormone concentrations in blood

The specific quantitative ELISA assay kits (Elabscience Biotechnology Inc., Houston, TX, USA) were used to measure the blood cortisol, insulin, and IGF-1 concentrations. All the measurements were assessed in line with the instructions provided in the kit, using the SPECTROstar® Nano microplate spectrophotometer, BMG Labtech in Germany.

### 2.6 Statistical analysis

Statistical analysis was conducted using the GraphPad Prism 9 software program (San Diego, CA, USA). Data were statistically evaluated through Unpaired t test with Welch correction. Significance levels were set at *p* < 0.05, *p* < 0.01, *p* < 0.001, and *p* < 0.0001 to assess differences between the cows with retained fetal membranes and the ones with normal parturition. All determinations were carried out using the two-stage step-up method (Benjamini, Krieger, and Yekutieli), and the results were presented as the mean values ± standard deviations.

## 3 Results and discussion

### 3.1 Blood metabolic profile

**Figure 1:**
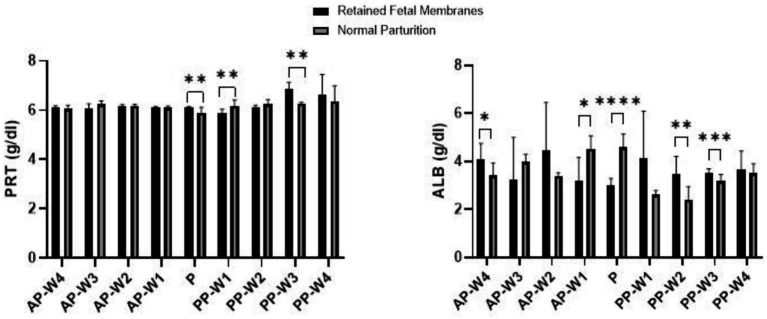
Total protein and albumin levels. Statistical significance *p < 0.05, **p < 0.01, ***p < 0.001, ****p < 0.001. The outcomes represent mean ± SD of three replicate analyses.

The findings of the study regarding total proteins and albumins during the prepartum, parturition, and postpartum periods are displayed in Figure 1. In the prepartum phase, from AP-W4 to AP-W1, no statistically significant differences were observed between the RFM and normal parturition (NP) groups. This suggests that any alterations in protein levels in cows that later developed RFM are either not present or not detectable until parturition.

During parturition (P), we observed a statistically significant elevation in protein levels in the RFM group compared to the NP group, surpassing the threshold for strong significance (*p* < 0.01). This period appears to be a critical point; physiological parameters are significantly modified in cows with RFM.

The immediate postpartum period presented a dynamic shift in protein levels. At PP-W1, the protein levels in cows with RFM were significantly lower than those in the NP group (*p* < 0.01), indicating a continuation of the significant physiological differences noted at parturition. However, by PP-W2, the significance of these differences decreased (*p* < 0.05), hinting at a possible transitional recovery phase. A notable spike in protein levels was observed in the RFM group at PP-W3 which not only re-established the strong significance seen during parturition but also reached a very high level of significance (*p* < 0.001). This could imply a delayed or sustained response to the physiological stress of RFM, with potential implications for the cow’s metabolic state and recovery. By PP-W4, the differences in protein levels between the two groups were no longer statistically significant (*p* > 0.05), suggesting a return to homeostatic levels or an adaptation to postpartum conditions. The lack of significant difference at this stage may indicate that the initial postpartum disturbances have resolved or that compensatory mechanisms have effectively mitigated the metabolic discrepancies associated with RFM.

During the prepartum phase, the albumin levels show statistically significant differences at 1 week before parturition (*p* < 0.05) indicating that changes in albumin levels associated with RFM become more pronounced as parturition approaches. Interestingly, the albumin levels at 4 weeks before parturition also demonstrate significant differences (*p* < 0.05), albeit the effect is less pronounced than at AP-W1. This suggests that while albumin levels begin to diverge between RFM and NP groups several weeks before parturition, the most substantial alterations occur closer to parturition.

The point of parturition (P) itself marks a highly significant decrease in albumin levels in RFM cows compared to NP cows (*p* < 0.0001), reflecting a major physiological impact during this critical time. This acute change likely represents a combination of stress responses and the metabolic demands placed on the liver, which is the primary site of albumin synthesis.

Postpartum albumin levels indicate a return toward normalcy but with some fluctuations. Immediately after parturition (PP-W1), the difference in albumin levels is not statistically significant (*p* > 0.05). However, by the second week postpartum (PP-W2), there is a significant increase in albumin levels in the RFM group (*p* < 0.01), suggesting a rebound effect or a delayed response to the physiological events of parturition. By the third week postpartum (PP-W3), albumin levels in RFM cows are again significantly higher than in NP cows (*p* < 0.001), indicating ongoing physiological adjustments or the continued effects of RFM. Finally, by the fourth week postpartum (PP-W4), the albumin levels are not significantly different between the two groups (*p* > 0.05), which may indicate a stabilization of albumin levels as cows recover from RFM.

**Figure 2:**
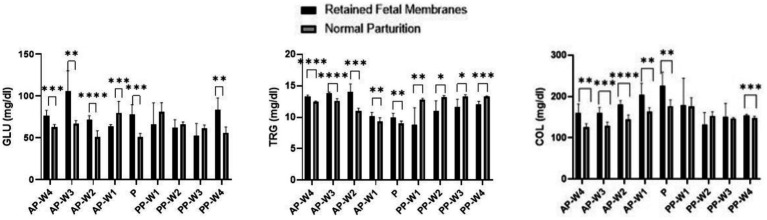
Glucose, triglycerides and total cholesterol levels. Statistical significance *p < 0.05, **p < 0.01, ***p < 0.001, ****p < 0.001. The outcomes represent mean ± SD of three replicate analyses.

Data on the variations in glucose, triglyceride, and total cholesterol concentrations during parturition and the antepartum and postpartum periods are presented in Figure 2.

In the weeks leading up to parturition, there were remarkable differences in glucose levels between the two groups. Starting at 4 weeks prior to parturition (AP-W4), cows with RFM exhibited significantly higher glucose levels than NP cows (*p* < 0.001), which persisted into the second week (AP-W2) before parturition. These findings may suggest an altered metabolic state or an increased stress response in RFM cows, which ultimately contribute to the development of such pathology. However, 3 weeks before parturition (AP-W3), RFM cows had extraordinarily high glucose levels (*p* < 0.01), which could imply an acute stress response or a compensatory mechanism against metabolic demands. Intriguingly, 1 week before parturition (AP-W1), RFM cows showed significantly lower glucose levels than NP cows (*p* < 0.001), indicating a possible metabolic shift or the onset of energy depletion as parturition approached. At the point of parturition (P), glucose levels in RFM cows were significantly higher (*p* < 0.001) compared to NP cows. The marked elevation in glucose could reflect the intense energy requirements and stress associated with RFM cows. This is a critical finding as it underscores the metabolic challenge faced by cows with RFM during labor, which may have implications for their health and recovery postpartum.

Immediately after parturition (PP-W1), the difference in glucose levels was not statistically significant (*p* > 0.05), which may suggest an initial period of recovery where glucose levels begin to stabilize. However, the subsequent weeks (PP-W2 and PP-W3) continued to show no significant differences (*p* > 0.05), indicating that the metabolic disturbance observed prepartum may resolve or diminish in the early postpartum period. Notably, by the fourth week postpartum (PP-W4), RFM cows demonstrated a significant increase in glucose levels (*p* < 0.01), which might indicate a delayed response to the metabolic demands or a secondary stress event.

In the antepartum period, the data indicate a statistically significant elevation in triglyceride levels in cows with RFM when compared to NP cows. This elevation is evident from 4 weeks before parturition (AP-W4) with a *p*-value <0.0001 and becomes more pronounced 3 weeks before parturition (AP-W3) with a *p*-value <0.0001. This suggests a substantial alteration in the lipid metabolism of RFM cows well before parturition, potentially due to increased mobilization of fat reserves or a stress-related increase in lipolysis. By 2 weeks prior to parturition (AP-W2), the difference peaks significantly (*p* < 0.001), indicating the highest disparity in triglyceride levels between the two groups during the prepartum period. Interestingly, 1 week before parturition (AP-W1), while the levels remain significantly higher in RFM cows (*p* < 0.01), the difference diminishes somewhat, which could suggest the beginning of a metabolic adjustment as parturition approaches. At the time of parturition (P), RFM cows continue to exhibit significantly higher triglyceride levels (*p* < 0.01) compared to NP cows. This persistent hypertriglyceridemia may reflect the ongoing metabolic stress or an inflammatory state associated with RFM, which could have implications for the cow’s energy status and the health of the neonate. Following parturition, the pattern reverses; the RFM group shows significantly lower triglyceride levels than the NP group from the first week postpartum (PP-W1, *p* < 0.01) through to the fourth week postpartum (PP-W4, *p* < 0.001). This decline could be indicative of a higher postpartum energy deficit in RFM cows, possibly due to a more extensive use of fat reserves for energy production or due to a reduced feed intake. The significant drop in triglycerides during the postpartum period may also signal a risk for the development of metabolic disorders, such as fatty liver syndrome, which is a concern in dairy cattle management.

Significant elevations in cholesterol were observed in RFM cows as early as 4 weeks before parturition (AP-W4), with a *p*-value <0.01, which may suggest an altered lipid metabolism or an increase in mobilization of fat reserves as part of the body’s preparation for the energy demands of parturition. This elevation persisted through the third week (AP-W3, *p* < 0.001) and peaked at the second week (AP-W2, *p* < 0.0001) before parturition, indicating the highest level of dyslipidemia in the prepartum period. The continued significant difference 1 week before parturition (AP-W1, *p* < 0.01) adds to the evidence that RFM is associated with marked changes in cholesterol metabolism leading up to parturition. At the point of parturition (P), there was a notable peak in cholesterol levels in RFM cows (*p* < 0.01), suggesting that the physiological stress of parturition may exacerbate the hypercholesterolemia. This could reflect the acute metabolic demands placed on the liver for cholesterol, which is vital for hormone synthesis and cellular functions that are critical during parturition. Immediately after parturition (PP-W1), the difference in cholesterol levels between RFM and NP cows was not statistically significant (*p* > 0.05), which may indicate a transient return to baseline levels or effective management of cholesterol following parturition. However, by the fourth week postpartum (PP-W4), a significant difference re-emerged (*p* < 0.001), suggesting a possible delayed response in the cholesterol metabolism of RFM cows or a secondary stress response as the cows continue to recover. The non-significant findings at PP-W2 (*p* > 0.05) and PP-W3 (*p* > 0.05) indicate no substantial differences in cholesterol levels between the two groups during this period. It is possible that the lipid metabolism is stabilizing during this time, or the cows may have adapted to the postpartum state regardless of the complications associated with RFM.

**Figure 3:**
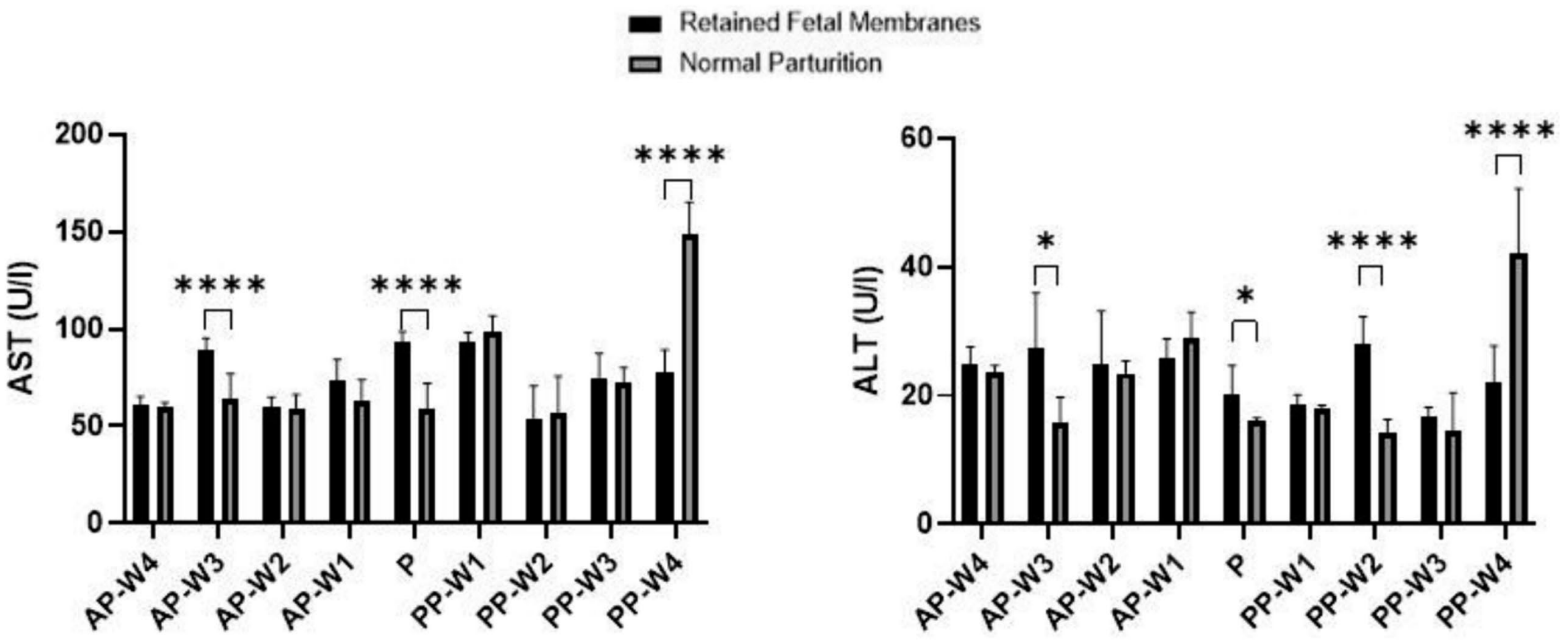
AST and ALT activity. Statistical significance *p < 0.05, ****p < 0.001. The outcomes represent mean ± SD of three replicate analyses.

Figure 3 displays the results of the blood transaminase activity (ALT and AST) assays. Leading up to parturition, we observe no statistically significant differences in AST levels at 4 weeks (AP-W4, *p* > 0.05) and 2 weeks (AP-W2, *p* > 0.05) before parturition. However, a significant spike in AST activity is noted at 3 weeks before parturition (AP-W3, *p* < 0.0001), suggesting an early onset of cellular stress or damage in cows that would later exhibit RFM. The reason for this elevation is not immediately apparent but could be indicative of the metabolic and physiological changes preparing the cows for the upcoming parturition. At 1 week before parturition (AP-W1), the increase in AST activity approaches significance (*p* < 0.05), possibly heralding the impending stress of labor. At the time of parturition (P), there is a highly significant elevation in AST levels in RFM cows (*p* < 0.0001), which could be attributed to the substantial physiological strain of parturition itself. The elevated AST levels could reflect increased cellular turnover or damage, potentially as a result of hypoxia or inflammation related to RFM.

Interestingly, the postpartum period shows no significant differences immediately after parturition (PP-W1 PP-W2, PP-W3, *p* > 0.05). This lack of significant difference might indicate that the acute stress on tissues typically resolves or diminishes after delivery. However, by the fourth week postpartum (PP-W4), there is a profound and highly significant decrease in AST levels in RFM cows (*p* < 0.0001). This decrease could suggest a resolution of the cellular stress or a possible compensatory mechanism after the initial postpartum period. The significant decrease in AST at PP-W4, especially considering the high levels during parturition, may also point to a substantial recovery or normalization process that extends into the postpartum period. This could be reflective of the liver and muscle tissues’ repair and regeneration capabilities.

The data indicate no significant difference in ALT levels between RFM and NP cows during the majority of the prepartum period, with non-significant *p*-values at AP-W4, AP-W2 and AP-W1 (*p* > 0.05). However, there is a notable exception at 3 weeks before parturition (AP-W3), where a significant increase in ALT levels in RFM cows (*p* < 0.05) suggests an early onset of hepatic stress or subclinical liver injury that may be related to the metabolic changes preceding parturition. At the time of parturition (P), a modest increase in ALT levels is observed (*p* < 0.05). While not highly significant, this increase aligns with the physiological strain of labor and could be indicative of mild liver stress or increased muscular activity associated with parturition efforts. In the immediate postpartum period (PP-W1), ALT levels show no significant difference (*p* > 0.05), suggesting no sustained hepatic insult following parturition. However, by the second week postpartum (PP-W2), there is a significant elevation in ALT levels in RFM cows (*p* < 0.0001), which could be indicative of ongoing liver stress or a delayed response to the metabolic challenges posed by RFM. By the third week postpartum (PP-W3), ALT levels are not significantly different (*p* > 0.05), potentially signaling a trend toward normalization. Yet, by the fourth week postpartum (PP-W4), there is a highly significant decrease in ALT levels in RFM cows (*p* < 0.0001), which is a somewhat unexpected finding. This decrease may reflect a resolution of earlier hepatic strain or a compensatory adaptation after the initial postpartum period. However, the lower levels of ALT could also raise concerns about the liver’s functional capacity, considering that such a significant decrease might also imply a reduction in hepatocellular mass or function.

### 3.2 Blood hormonal profiles

**Figure 4:**
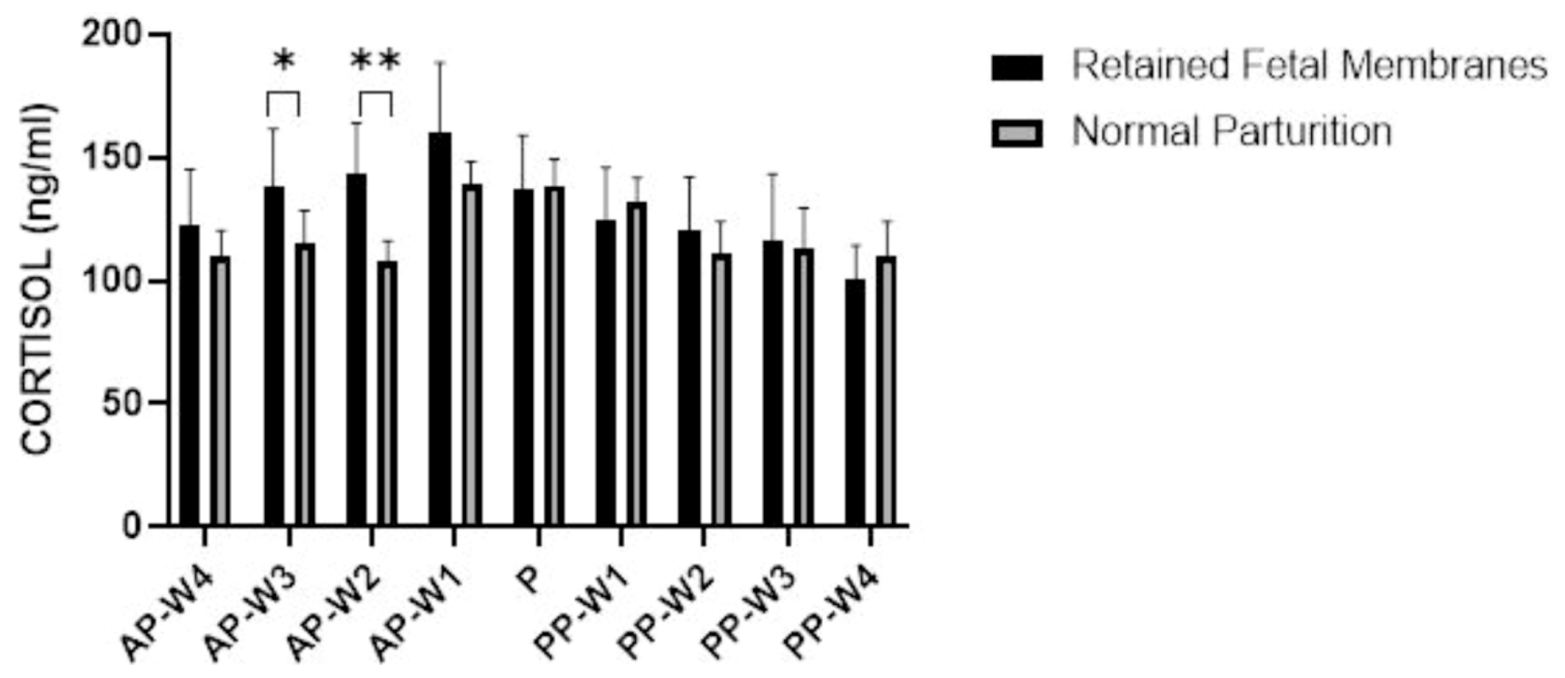
Blood cortisol concentrations. Statistical significance *p < 0.05, **p < 0.01. The outcomes represent mean ± SD of three replicate analyses.

The data suggest that cortisol levels in cows with RFM are not significantly higher than those with NP at 4 weeks before parturition (AP-W4, *p* > 0.05), suggesting that at this stage, stress levels do not differ markedly between the two groups. However, a trend begins to emerge 3 weeks’ prior (AP-W3, *p* < 0.05) and becomes significant 2 weeks before parturition (AP-W2, *p* < 0.01), with RFM cows exhibiting higher cortisol levels. This could indicate an increasing stress response or a physiological preparation for the upcoming labor, which may be more pronounced in cows predisposed to RFM. One week prior to parturition (AP-W1), the difference in cortisol levels is not significant (*p* > 0.05), which might suggest a complex interplay of factors as the cow’s approach labor. Interestingly, at the time of parturition (P), the data do not show a significant difference in cortisol levels between the two groups (*p* > 0.05). This could be due to the high variability of cortisol responses during labor or a possible equalization of stress levels between RFM and NP cows induced by the parturition process itself. In the postpartum period, the cortisol levels do not show significant differences between RFM and NP cows at any week (PP-W1 to PP-W4), with *p*-values >0.05. This lack of significant variation postpartum suggests that the stress associated with RFM may not extend beyond parturition in a way that is reflected in systemic cortisol levels, or that both groups of cows experience a normalization of cortisol levels as they recover from parturition. However, it is noteworthy that the cortisol levels in RFM cows significantly decrease by the fourth week after parturition (PP-W4, *p* > 0.05), approaching significance. This decrease may reflect a return to baseline stress levels or successful adaptation to postpartum life. Figure 4 shows a detailed replication of these outcomes.

**Figure 5:**
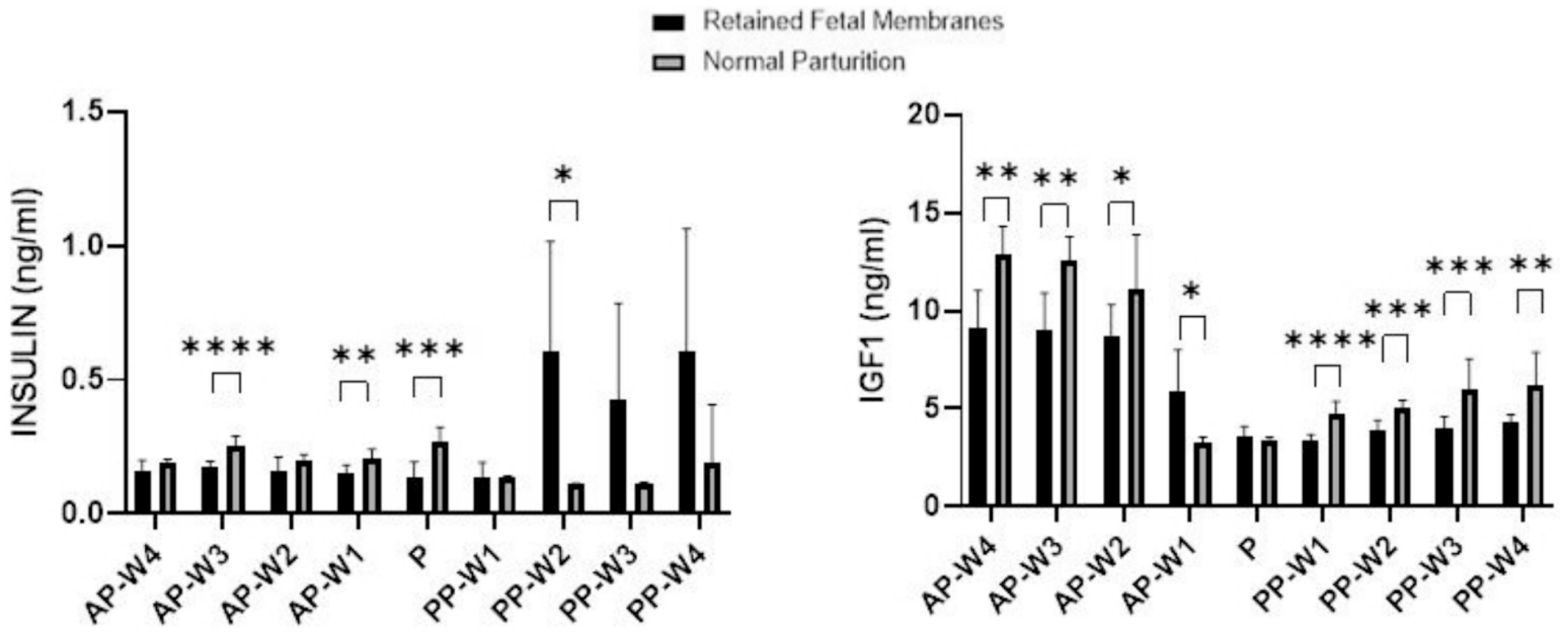
Blood Insulin and IGF-1 concentrations. Statistical significance *p < 0.05, **p < 0.01, ***p < 0.001, ****p < 0.001. The outcomes represent mean ± SD of three replicate analyses.

Figure 5 displays the data obtained following the evaluation of blood insulin and IGF-1 concentrations during antepartum period, parturition and postpartum period.

During the prepartum phase, there were no significant differences in insulin levels between RFM and NP cows 4 weeks before parturition (AP-W4, *p* > 0.05) and 2 weeks before parturition (AP-W2, *p* > 0.05). However, a significant decrease in insulin levels was observed 3 weeks before parturition (AP-W3, *p* < 0.0001) in cows with RFM. This suggests an early metabolic alteration in these cows, potentially indicative of a stress response or altered glucose metabolism. Additionally, 1 week before parturition (AP-W1), a significant difference is noted again (*p* < 0.01), which could signify the continuation or amplification of these metabolic changes as parturition approaches. At the time of parturition (P), a significant reduction in insulin levels is observed in cows with RFM (*p* < 0.001). This notable decrease may reflect a strong stress response or an energy deficit due to the increased demands of labor, which could be exacerbated in cows that go on to develop RFM. Immediately following parturition (PP-W1), insulin levels do not differ significantly between the two groups (*p* > 0.05), suggesting a return to baseline or a resolution of the acute parturition-related metabolic disturbances. However, the second week postpartum (PP-W2) shows a significant increase in insulin levels in RFM cows (*p* < 0.05). This might reflect a postpartum metabolic adaptation or a rebound from the energy deficits incurred during parturition. By the third week postpartum (PP-W3) and the fourth week postpartum (PP-W4), although the differences in insulin levels are not statistically significant (*p* > 0.05), there is a trend toward higher insulin levels in RFM cows. This could indicate a persistent alteration in glucose metabolism or insulin sensitivity in these cows, which may have long-term implications for their health and productivity.

Significant decreases in IGF-1 levels were observed in cows with RFM compared to NP cows as early as 4 weeks before parturition (AP-W4, *p* < 0.01), and these persisted through to 3 weeks (AP-W3, *p* < 0.01) and 2 weeks (AP-W2, *p* < 0.05) before parturition. This trend suggests a possible alteration in the metabolic state of RFM cows, which could be due to a higher energy demand or a stress response related to the impending parturition. Intriguingly, 1 week before parturition (AP-W1), RFM cows showed an increase in IGF-1 levels compared to NP cows (*p* < 0.05), which could indicate a compensatory mechanism or a distinct shift in metabolic focus as parturition approaches. At the time of parturition (P), no significant difference was found in IGF-1 levels between RFM and NP cows (*p* > 0.05). This could be due to the acute and possibly equal physiological stress of parturition on all animals, overshadowing the subtler chronic stressors that may differentiate RFM from NP cows. Immediately following parturition (PP-W1), a significant decrease in IGF-1 levels was detected in RFM cows (*p* < 0.0001). This suggests a continuation of the energy deficit or a more pronounced response to the metabolic and physiological stresses of RFM. The significant differences continue into the second and third weeks postpartum (*p* < 0.001). By the fourth week postpartum (PP-W4), IGF-1 levels in RFM cows were still significantly lower than NP cows (*p* < 0.01), which could reflect a longer-term impact of RFM on the metabolic health of the cows, possibly influencing recovery and subsequent productivity.

## 4 Discussion

There is a notable increment in serum total protein levels in bovines commencing 2 months prior to the expected parturition date, reaching a zenith 1 month before parturition, followed by a swift decrement approaching the parturition period (20). This pattern underscores the translocation of immunoglobulins from the serum to the mammary gland, initiating several weeks before parturition and attaining maximum concentrations between 1 to 3 days before the (20, 21).

The typical plasma concentration range of albumin in bovines is established to be 3.03 to 3.55 g/dL (22). Albumin is classified as a negative acute-phase protein, characterized by a gradual decrement in its concentration, with more significant reductions observed in the context of chronic inflammatory disorders. Negative acute-phase proteins are delineated as serum proteins that exhibit a decrease in concentration of 25% or greater during the acute phase in response to infection, inflammation, or trauma (20). Trevisi et al. (23) documented the inflammatory alterations following a retained placenta, indicating that cows exhibit diminished albumin levels during the postpartum period (Day 1–28.3 g/L; Day 7–28.3 g/L; Day 14–28.6 g/L; Day 21–29.4 g/L; Day 28–30.3 g/L) in comparison to their healthy counterparts (Day 1–34.2 g/L; Day 7–32.1 g/L; Day 14–32.1 g/L; Day 21–32.8 g/L; Day 28–33.4 g/L). This reduction is attributed to compromised hepatic function and a deteriorated energy balance. Corresponding observations were presented in the research by Semacan and Seminc (24), where bovines with a retained placenta demonstrated lower albumin levels (2.93 ± 0.07 g/100 mL) relative to those in a healthy condition (3.32 ± 0.07 g/100 mL). A typical decline in albumin levels immediately post-calving is noted (25, 26), which is also observed in cows undergoing normal calving, indicating an improved protein status at the organismal level.

The basal plasma concentration of total proteins in bovines is delineated within a range of 6.74 to 7.46 g/dL, as documented by Farver et al. (22). Investigations by Hashem and Amer (27) reveal an elevation in total protein concentrations in bovines afflicted with retained placenta (8.70 ± 0.44 mg/1 dL) in comparison to those undergoing uneventful parturition (8.41 ± 1.58 mg/1 dL). Conversely, research by Civelek et al. (28) indicates a reduction in total protein levels during parturition in cows with retained placenta (6.62 ± 0.13 mg/dL) relative to those with normal parturition processes (8.15 ± 0.36 mg/dL). Kumari et al. (29) corroborate these findings, noting significantly diminished total protein concentrations in bovines with retained placenta across multiple time points: 14 days, 7 days, and 5 days preceding parturition, during the parturition event itself, and on the first and second days postpartum, when compared to bovines experiencing normal parturition. In our studies, during parturition, we observed a significant elevation in protein levels in the RFM group compared to the NP group, this period appears to be a critical point; physiological parameters are significantly modified in cows with RFM. Three weeks after parturition, there was a noticeable increase in protein levels in the RFM group. This may indicate a prolonged or delayed reaction to the stress of RFM, which could have consequences for the cow’s recovery and metabolic state. The protein levels in the RFM group were not significantly different from the NP group by week 4 postpartum, indicating a return to physiologic values or an adaptation to postpartum conditions.

The normative plasma glucose concentration in bovines is quantified as ranging between 45 and 75 mg/dL, according to Farver et al. (22). Empirical data elucidate that bovines suffering from retained placenta manifest diminished glucose levels during the immediate aftermath of parturition compared to those experiencing normal delivery. Semacan and Sevinc (24) reported a significant disparity in blood glucose levels, with cows afflicted by retained placenta showing lower levels (61.6 ± 6 mg/dL) versus those in healthy conditions (88.9 ± 7.3 mg/dL). This trend of reduced glucose levels in cows with retained placenta relative to those undergoing normal calving is consistently observed across various studies (27, 28). Kumari et al. (29) established that bovines with retained placenta exhibited lower glucose concentrations on days 14, 7, 5, 3, 1 preceding calving, during the calving process, and on the first postpartum day in comparison to cows with uneventful deliveries. These findings are in accordance with the data that was obtained during our investigations. Compared to NP cows, RFM cows had significantly higher glucose levels at parturition (P). The high energy requirements and stress that RFM cows experience may be the cause of the significant increase in glucose.

The occurrence of hypoglycemia in bovines with retained placenta underpins the hypothesis that glucose concentrations toward the culmination of pregnancy act as an indicator of elevated risk for retained placenta. An alternative explanation posits that the decreased glucose levels may be attributed to augmented nutritional demands of the fetus and the formation of colostrum (27). Furthermore, hypoglycemia might be associated with elevated cortisol levels related to retained placenta and postpartum metritis, either in a direct or indirect manner (27, 30). The presence of hypoglycemia during the final month of gestation is identified as a risk factor for the development of retained placenta and postpartum metritis (27, 31).

Additionally, the increased susceptibility to retained placenta is linked to hypotonia or atonia within the musculature of the reproductive and digestive tracts, a condition precipitated by diminished concentrations of glucose and calcium within the smooth muscle cells (7, 32).

The typical plasma concentration range for triglycerides in bovines is reported to be 0 to 14 mg/dL (22). Kaczmarowski et al. (30) posited that diminished lipid and triglyceride levels might result from altered lipid metabolism, augmented activity of tissue lipolytic enzymes, or ketosis in afflicted animals, with ketone bodies measured at 765 ± 427 μmol/L in cows with retained placenta versus 754 ± 296 μmol/L in their healthy counterparts. A lower amount of triglycerides was observed throughout our research, which may indicate that RFM cows have a greater postpartum energy deficit. In addition, the significant decrease in triglycerides during the postpartum phase may also indicate a risk for the development of metabolic disorders (such fatty liver syndrome).

Deviations in triglyceride concentrations are frequently linked to hepatic disorders, as illustrated by Semacan and Sevinc (24), where bovines with retained placenta exhibited lower triglyceride levels (15.57 ± 1.4 mg/100 mL) in comparison to healthy bovines (21.5 ± 1.6 mg/100 mL). Fatty liver infiltration or hepatic steatosis, which can significantly impair hepatic function, is one such disorder. A notable effect of hepatic steatosis includes a reduction in serum albumin levels, indicative of compromised liver synthetic capacity, and an increase in plasma enzyme activity such as AST, aligning with observations made in this discourse.

The normative plasma cholesterol range in bovines is delineated as 80 to 120 mg/dL, according to Farver et al. (22). It has been observed that cows with retained placenta demonstrate significantly elevated plasma cholesterol levels compared to those that efficiently expel fetal membranes. Moreover, decreased circulating cholesterol levels during the antepartum period have been associated with a heightened risk of retained placenta. This trend is corroborated by Semacan and Sevinc (24), where cows with retained placenta had cholesterol levels of 96.1 ± 6.8 mg/100 mL, whereas healthy cows had 141.3 ± 9.8 mg/100 mL. Similar findings were echoed in additional studies comparing healthy cows to those with retained placenta (25, 27, 28). In our study, significant increases in cholesterol have been observed in RFM cows antepartum. The RFM cows’ cholesterol levels peaked at parturition (P), indicating that the physiological stress associated with parturition might exacerbate the hypercholesterolemia.

On the other hand, Kaczmarowski et al. (30) found no statistically significant differences in cholesterol levels between cows with retained placenta and healthy ones (2.08 ± 0.83 mmol/L vs. 2.17 ± 0.83 mmol/L). Kumari et al. (29) reported lower cholesterol levels in cows with retained placenta on day 1 antepartum, during parturition, and on day 1 postpartum. Blood cholesterol concentration serves as a vital marker for hepatic lipoprotein synthesis and the availability of exogenous energy. Despite cholesterol levels surpassing physiological norms in both groups, cows with retained placenta exhibited higher cholesterol levels compared to those without postpartum pathologies. Blood cholesterol content thus acts as a crucial indicator of hepatic lipoprotein synthesis and external energy availability (29), which in turn reflects on liver health.

The standard enzymatic activity range for Aspartate Aminotransferase (ASAT) in bovines is identified as 78 to 132 U/L, as established by Farver et al. (22). Elevated ASAT levels observed in this study correlate with the known association between hepatic steatosis (fatty liver) and cows experiencing retained placenta, as highlighted by Semacan and Sevinc (24). The infiltration of fat within the liver is associated with an escalation in hepatic enzyme activities alongside a decrease in blood glucose, total lipids, cholesterol, and triglycerides. The spectrum of liver fat accumulation, from mild to severe, can precipitate liver dysfunction without leading to hepatocyte death, albeit with an increase in hepatic enzyme activity (24, 27). Semacan and Sevinc (24) attribute the accumulation of lipids within hepatocytes of cows with retained placenta to the presence of endotoxins stemming from infectious diseases such as endometritis, which can induce hepatic lesions and necrosis, thereby causing varying degrees of liver dysfunction.

AST activity, which is inherently high in the liver of all domestic species, typically surges in serum levels during both acute and chronic liver damages. Given that AST is also abundant in muscles, kidneys, pancreas, and erythrocytes, damage to these tissues can likewise result in elevated serum AST levels (33), as observed in cows with normal calving during the fourth postpartum week.

The normative range for ALAT activity in bovines is set at 11–40 U/L (22). Significant increases in ALT activity are typically indicative of hepatocellular inflammation. In such scenarios, a progressive reduction in ALT activity might signal recovery, with a decrease of 50% or more in serum ALT activity over several days being viewed as a positive prognostic indicator. However, some animals with severe liver disease may present with normal serum ALT levels, and a reduction in serum ALT activity could signify a substantial loss of viable hepatocytes or a diminished synthesis of transaminases (33).

The induction of corticotropin and adrenocorticotropic hormone production serves as a pivotal mechanism activating the hypothalamic–pituitary axis, which in turn elevates plasma cortisol levels significantly from a normative baseline of approximately 5 ng/mL to a range of 10–20 ng/mL (7, 34). This surge in cortisol, recognized for its immunosuppressive capabilities, notably impairs leukocyte proliferation and their critical functional roles, effectively obstructing the standard immunological processes essential for the identification and subsequent elimination of fetal tissues (7, 13).

Pre-parturition cortisol levels experience a marked increase, reaching their zenith during or shortly subsequent to the birth process. This elevation of cortisol plays a multifaceted role during parturition; it may exert a negative feedback mechanism on prostaglandin synthesis via the induction of lipocortin-1, a phospholipase inhibitor, or alternatively, it may contribute to the stress-induced labor process (35).

Normative plasma cortisol concentrations in cattle are delineated to range between 1.3 and 2.93 ng/mL (22). However, during the periparturient phase, a compromised immune system is observed, largely attributed to metabolic stress precipitated by hormonal and metabolic fluctuations, a negative energy balance, and deficiencies in proteins, minerals, and essential vitamins necessary to meet fetal demands and support the initiation of lactation (36). This metabolic stress is capable of activating the hypothalamic–pituitary-adrenocortical (HPA) axis, leading to an augmented secretion of plasma corticosteroids. As a result, there is a dramatic elevation in cortisol levels during the periparturient phase, notably on the day of calving. Cortisol, with its potent immunosuppressive properties, suppresses the proliferation and function of leukocytes during periods of stress, significantly reducing neutrophil phagocytosis, lymphocyte cytotoxic capacity, and cytokine activity. These reductions compromise the efficacy of immunological recognition and the expulsion of fetal membranes, thereby contributing to incidences of retained placenta (36). Cortisol levels 4 weeks prior to parturition in cows with RFM do not appear to be substantially higher than in cows with NP, according to our data. However, RFM cows presented increased cortisol levels 2 weeks prior to parturition. These modifications could indicate an increasing stress response or a physiological preparation for the upcoming labor, which may be more pronounced in cows predisposed to RFM.

The literature suggests that the average insulin concentration during the periparturient period spans from 0.2 ng/mL to 1.6 ng/mL (37). Moreover, periparturient hypocalcemia is intricately linked to metabolic activities, where diminished calcium levels adversely affect insulin production, thereby reducing glucose delivery to various tissues (7, 14). This deficiency in glucose uptake triggers lipolysis, leading to a negative energy balance, metabolic stress, immunosuppression, and ketosis. The resultant hypotension or atony of the reproductive and digestive tract musculature, owing to reduced glucose and calcium levels within smooth muscle cells, heightens the risk of retained placenta (7, 32).

Comparative analyses reveal that insulin levels in bovines with normal calving are higher during the antepartum and calving phases in contrast to those with retained placenta, albeit lower across all sampling points than the literature average (37).

Furthermore, the mean concentration of insulin-like growth factor 1 (IGF-1) across various phases has been documented by Baldacim et al. (38), ranging from 8.67–11.62 ng/mL in the antepartum period to 2.50–4.50 ng/mL postpartum. Cows experiencing normal calving exhibit higher IGF-1 levels in the prepartum period as compared to those with retained placenta. Notably, during calving, the IGF-1 levels are comparable across both groups, with a subsequent increase observed in cows with normal calving relative to those with retained placenta.

Postpartum, a decrease in IGF-1 levels has been observed (39). Velazquez et al. (10) describe a pattern wherein IGF-I concentrations diminish in the final days of pregnancy, reach a low point in the initial weeks post-calving, and subsequently commence an upward trajectory.

The IGF-1 levels in cows undergoing normal calving remain within physiological parameters during both the antepartum and postpartum periods and exhibit a more rapid recovery. In contrast, cows with retained placenta showcase lower IGF-1 levels in the initial postpartum week than their healthy counterparts, falling beneath the concentration range specified in the literature (38). Nakada (12) associates lower IGF-I levels with retained placenta and endometritis, noting that IGF-I concentrations decline temporarily in cows suffering from postpartum reproductive disorders and seldom revert to baseline levels post-lactation cessation.

## 5 Conclusion

In conclusion, this clinical study reveals the multifaceted metabolic challenges faced by cows with retained fetal membranes (RFM) across various biochemical parameters during the periparturient period. Our findings elucidate the temporal complexity of metabolic alterations, including significant impacts of albumin, glucose, triglyceride, cholesterol levels, enzyme activities (AST, ALT), cortisol, insulin, and IGF-1 dynamics, highlighting the intricate interplay of metabolic stressors associated with RFM. Notably, the absence of significant prepartum differences in some parameters contrasts with marked postpartum fluctuations, indicating a critical period of metabolic adjustment and recovery following parturition. The significant prepartum and postpartum changes in these biochemical markers not only provide insights into the metabolic demands placed on cows by RFM but also offer potential diagnostic and management strategies to mitigate its impact. By closely monitoring these indicators, particularly during the critical weeks surrounding parturition, interventions can be targeted more effectively to support cow health, enhance recovery processes, and ultimately improve dairy herd productivity.

## Data availability statement

The original contributions presented in the study are included in the article/supplementary material, further inquiries can be directed to the corresponding author.

## Ethics statement

The animal study was approved by University of Agricultural Sciences and Veterinary Medicine Cluj-Napoca, Romania Bioethical Committee (256/21.04.2021) and the Regional Sanitary Veterinary and Food Safety Authority (no. 274/12.11.2021). The study was conducted in accordance with the local legislation and institutional requirements.

## Author contributions

HR: Conceptualization, Investigation, Methodology, Software, Visualization, Writing – original draft. IO: Methodology, Resources, Writing – original draft. OC: Investigation, Methodology, Writing – original draft. AM: Investigation, Writing – original draft. DD: Investigation, Writing – original draft. CȘ: Investigation, Writing – original draft. DN: Investigation, Writing – original draft. AB: Investigation, Writing – original draft. SA: Conceptualization, Data curation, Resources, Supervision, Validation, Visualization, Writing – review & editing.
